# GABARAP ameliorates IL-1β-induced inflammatory responses and osteogenic differentiation in bone marrow-derived stromal cells by activating autophagy

**DOI:** 10.1038/s41598-021-90586-9

**Published:** 2021-06-02

**Authors:** Xiaobo Guo, Zhenyuan Wu

**Affiliations:** Department of Orthopedics, Jincheng General Hospital, Jincheng, 048000 China

**Keywords:** Stem cells, Mesenchymal stem cells, Stem-cell differentiation

## Abstract

Bone mesenchymal stem cells (BMSCs) are the most commonly investigated progenitor cells in bone defect repair and osteoarthritis subchondral bone regeneration; however, these studies are limited by complex inflammatory conditions. In this study, we investigated whether pro-autophagic γ-aminobutyric acid receptor-associated protein (GABARAP) promotes BMSCs proliferation and osteogenic differentiation by modulating autophagy in the presence or absence of interleukin-1 beta (IL-1β) in vitro. The expression levels of all relevant factors were evaluated by qRT-PCR or western blotting where appropriate. BMSCs differentiation were assessed by Alizarin Red, alkaline phosphatase, safranin O, and Oil Red O staining. Furthermore, the interactions between autophagy and osteogenic differentiation were investigated by co-treatment with the autophagy inhibitor 3-methyladenine (3-MA). As the results, we found that treatment with recombinant human His6-GABARAP protein promoted cell proliferation, inhibited apoptosis, and reduced ROS generation by increasing autophagic activity, particularly when co-cultured with IL-1β. Moreover, His6-GABARAP could effectively increase the osteogenic differentiation of BMSCs. The expression levels of inflammatory factors were significantly decreased by His6-GABARAP treatment, whereas its protective effects were attenuated by 3-MA. This study demonstrates that GABARAP maintains BMSCs survival and strengthens their osteogenic differentiation in an inflammatory environment by upregulating mediators of the autophagy pathway.

## Introduction

Mesenchymal stem cells (MSCs), including BMSCs, display a robust proliferative capacity and multipotency that allows them to differentiate into adipocytes^1^, chondrocytes^2^, osteoblasts^3^, and other non-mesodermal lineage cells^4^ in different microenvironments. Therefore, these cells are considered promising therapeutic candidates for tissue engineering and display potential for clinical application in conditions such as osteoarthritis (OA). Previous studies have strongly indicated that BMSCs can effectively promote cartilage erosion regeneration in OA by not only differentiating into cartilage-forming chondrocytes but also modulating the behavior of subchondral bone^5^. However, the abnormal microenvironment of OA characterized by stress conditions, such as inflammation^6^ and oxidative stress^7,8^, reduces the survival capacity of BMSCs. Moreover, high pro-inflammatory cytokine levels inhibit BMSCs osteogenesis^9^. Therefore, approaches to promote BMSCs osteogenic differentiation and improve their cell survival under severe stress is particularly important for tissue renewal and subsequent regeneration^10^.

IL-1β is a primary initiator of inflammatory progression in OA^11^, with studies reporting that elevated IL-1β levels play a central role in inflammation-induced bone destruction^12,13^. Indeed, IL-1β expression levels are significantly higher in OA than in healthy sites and is associated with subchondral destruction^14^. Mechanistically, the inhibitory effects of IL-1β have been associated with MSCs proliferation and the activation of osteoclastogenesis^15,16^. IL-1β treatment can also dramatically induce the production of ROS, nitric oxide (NO), and proteolytic enzymes such as matrix metalloproteinase (MMP) during bone formation, thus inhibits BMSCs osteogenic differentiation and bone tissue generation in vitro and in vivo^17–19^. Although anti-inflammation has been studied for decades, our understanding of the mechanism underlying the effects of IL-1β on cell viability and osteoblast differentiation remains poor.

Previous studies have shown that the maintenance of bone mass stability is associated with the activation of cellular autophagy^20^, which is a highly conserved catabolic process that maintains cellular homeostasis and recycles degraded cytoplasmic materials^21–23^. Autophagy can also maintain MSCs stemness and protect cells against stress pathology signals, including ROS, inflammation, and metabolic precursors^24,25^; for instance, inhibiting autophagy in osteoblast‐like cells increases oxidative stress and stimulates their apoptosis^26^. During osteogenesis, autophagy is critical for decreasing osteopenia by accelerating mineralization and osteogenesis in vitro and in vivo^20,27^, indicating that increased autophagy may maintain the BMSCs phenotype after IL-1β treatment and enable the promotion of osteoblast differentiation.

GABARAP is a member of the autophagy-related protein 8 (Atg8) family^28^ that displays a high degree of sequence homology with the autophagy marker light chain 3 (LC3). Kabeya et al.^29^ showed that GABARAP binds to autophagic vesicles in a similar manner to LC3 and may also be involved in autophagosome formation. By modulating autophagy, GABARAP has been reported play a key role in suppressing tumor growth^30^, inhibiting inflammation progression^31^, and regulating angiogenic activity^32^ via processes associated with vesicle transport, apoptotic cell death, and ROS generation. However, the role of GABARAP in regulating the differentiation fate of BMSCs via autophagy remains unclear.

Based on the studies described above, we hypothesized that the mediation of ROS generation and cellular autophagy by GABARAP may play pivotal roles in IL-1β-induced BMSCs injury. This study is the first to demonstrate the regulatory effects of GABARAP on IL-1β-induced cell apoptosis and BMSCs osteogenic differentiation in vitro, with a particular focus on the relationship of GABARAP with autophagy signaling pathways. Our long-term goal is to utilize GABARAP in bone regenerative therapy, such as improving the efficacy of BMSCs-based cellular regenerative therapies.

## Materials and methods

### BMSCs isolation and culture

Bone marrow was flushed from the femurs and tibias of 1–5-day-old New Zealand rabbits (Animal Resources Centre of Guangxi Medical University, Nanning, Guangxi, China) with α-modified Eagle’s medium (α-MEM; Gibco, Thermo Fisher Scientific, Waltham, MA, USA) containing 10% (v/v) fetal bovine serum (FBS; Hyclone, Logan, UT, USA) and 1% (v/v) penicillin/streptomycin (Solarbio, Beijing, China) using a 27-gage syringe. The cell suspension was strained through a 70 μm mesh filter and cultured in the same medium at 37 °C under 5% CO_2_. At 80–90% confluence, the cells were trypsinized, expanded, and used for different assays after their third passage. The animal protocol was approved by the Animal Ethical Committee of the Animal Resources Centre of Guangxi Medical University (ethic cord: 201902003). All animal experiments were performed in accordance with relevant guidelines and regulations. Meanwhile, animal studies were reported in compliance with the ARRIVE guidelines^33,34^ and complied with the principles of replacement, refinement and reduction (the 3Rs).

### BMSC multi-differentiation and cytological staining

Differentiated BMSCs were stimulated to undergo adipogenesis, osteogenesis, and chondrogenesis according to standard procedures^35–37^. Briefly, at 90% confluence, the BMSCs were incubated in a differentiation culture medium for a further 21 days. For adipocyte differentiation, the complete culture medium was supplemented with 1 mM (10 nM) dexamethasone (Sigma, St Louis, MO, USA), 100 nM (0.5 mM) indomethacin (Sigma), and 2 mM insulin (Sigma). Adipogenesis was verified by Oil Red O staining (Beyotime, Beijing, China). To induce osteogenic and chondrogenic differentiation, the BMSCs were cultured with osteogenic medium [10 mM β-glycerophosphate (Sigma), 50 mg/ml ascorbic acid 2-phosphate (Sigma), and 100 nM dexamethasone] or chondrogenic medium [50 μg/mL ascorbic acid 2-phosphate, 100 nM dexamethasone, 1% insulin-transferrin-selenium solution (Gibco), and 10 ng/mL TGF-β1 (PeproTech, Rocky Hill, PA, USA)], respectively. Alizarin Red (Sigma), ALP (Beyotime), and safranin O (Solarbio) staining were conducted to visualize calcium accumulation and evaluate chondrogenesis according to the manufacturer’s suggested protocol. All staining results were viewed using an upright microscope (Olympus, Japan).

### Inflammatory stimulation and the treatment of GABARAP and 3-MA in vitro

To determine the optimum His6-GABARAP (Boston Biochem, Cambridge, MA, USA) concentration for BMSCs proliferation, the cells were seeded in 96-well plates and treated with various concentrations of His6-GABARAP (0–200 nM) for 3 days. Viability was assessed using an MTT (3-(4,5-dimethylthiazol-2-yl)-2,5-diphenyltetrazolium bromide, Solarbio) assay, as described below. Based on the results, 30, 60, and 120 nM His6-GABARAP were used to assess the effects of GABARAP on cell proliferation, viability, intracellular ROS, and multi-differentiation.

To induce osteogenesis, BMSCs were cultured in osteogenic medium in the presence of three doses of His6-GABARAP (30, 60, and 120 nM) for 21 days, after which osteogenic differentiation was determined by Alizarin Red and alkaline phosphatase (ALP) staining. Based on the levels of intracellular calcium accumulation, 60 nM His6-GABARAP were selected as the most appropriate concentrations for observing the effect of His6-GABARAP on BMSCs osteogenic differentiation. Inflammation was stimulated by treating the cells with 10 ng/mL of IL-1β, after which the cells were treated with His6-GABARAP with or without the autophagy inhibitor 3-MA (5 mM, Sigma) to clarify its relationship with the autophagy pathway in conventional culture or osteogenic differentiation.

### Cell proliferation assay

BMSCs proliferation was assessed using MTT assays. One tenth of the volume of 5 mg/mL MTT solution was added to stimulated BMSCs per well and continuously incubated at 37 °C for 4 h in the dark. The solution was then discarded, 150 μL dimethyl sulfoxide (DMSO, Sigma) was added to each well, and the absorbance was measured at 490 nm using a Multiskan GO microplate reader (Thermo Fisher Scientific).

Hematoxylin and eosin (HE) staining: BMSCs cultured in 24-well plates were fixed for 30 min in 4% paraformaldehyde, stained with HE (Solarbio) according to the manufacturer’s instructions, and dehydrated using an ethanol gradient. The slides were then sealed and examined using a microscope (Olympus).

### Dual staining with fluorescein diacetate (FDA) and propidium iodide (PI)

Harvested BMSCs were incubated with 2 µM FDA (Invitrogen Life Technologies, CA, USA) and 2 µg/L PI (Invitrogen) for 5 min at 37 °C in the dark. Live and dead cells were simultaneously evaluated via laser scanning confocal microscopy (Nikon A1, Japan).

### Annexin V and PI staining

Differently treated BMSCs were harvested and re-suspended in Annexin V-FITC and PI working solutions (Thermo Fisher Scientific) and incubated at 4 °C in the dark. A total of 1 × 10^4^ cells per sample were acquired by flow cytometry (BD Biosciences), and the percentage of apoptotic and live cells was assessed using FlowJo software (Tree Star Inc. Ashland, OR, USA).

### Intracellular ROS measurement

Intracellular ROS were measured using a fluorescent 2,7-dichlorodihydrofluorescein diacetate (DCFH-DA) probe kit (Jiancheng) according to the manufacturer’s protocol. Briefly, suitably treated BMSCs were incubated with 10 μM DCFH‐DA at 37 °C for 30 min and fluorescence was assessed by flow cytometry and FlowJo software analysis.

### Monodansylcadaverine (MDC) staining

BMSCs were stained with 0.05 mM MDC (Sigma) to detect autophagic vacuoles according to the manufacturer’s instructions and assessed by laser scanning confocal microscopy, as described above.

### Transmission electron microscopy (TEM)

Harvested BMSCs were fixed for 24 h with 2.5% glutaraldehyde, incubated with 1% osmium tetroxide for 1 h in 4 °C, and stained with 2% uranyl acetate. The BMSCs were then dehydrated using an acetone gradient and embedded in araldite. Sample sections were cut, stained with toluidine blue, and observed by TEM (Hitachi, Tokyo, Japan).

#### Autophagy flux measurement

StubRFP-sensGFP-LC3 lentiviruses were constructed by Genechem Co. (Shanghai, China). Primary first passage BMSCs were seeded in 6-well plates, cultured overnight, and transduced with the lentivirus in serum-free medium at a multiplicity of infection of 50. After 12 h, the media was replaced with complete α-MEM and then the cells were treated with IL-1β, His6-GABARAP, and 3-MA. Autophagic flux was observed using a laser scanning confocal microscope (Nikon America Inc., Melville, NY). stubRFP and sensGFP punctae were counted manually in at least 40 cells per sample.

#### Total RNA isolation and quantitative RT-PCR (qRT-PCR)

Total RNA was isolated from BMSCs using Trizol reagent (Invitrogen), purified using a RNeasy mini kit (QIAGEN, Valencia, CA, USA), and reverse transcribed (1 mg total RNA per sample) using a Transcriptor First-strand cDNA synthesis kit (Roche, Basel, Switzerland). qRT-PCR was performed using SYBR green master mix on an Applied Biosystems 7500 Real Time Cycler (Applied Biosystems, CA, USA) under the following cycling conditions: 95 °C for 10 min, 40 cycles of 95 °C for 15 s and 60 °C for 1 min, followed by a standard melting curve. Samples were assessed in triplicate, with gene expression assessed using the 2^-ΔΔCT^ method and normalized to GAPDH. The primer pairs for the target genes are listed in Table 1.

**Table 1:** Primer sequences used in qRT-PCR experiments.

Gene	Primer	Primer sequence (5′–3′)
Glyceraldehyde-3-phosphate dehydrogenase (GAPDH)	Forward	AGACACGATGGTGAAGGTCG
	Reverse	TGCCGTGGGTGGAATCATAC
BCL2 associated X (BAX)	Forward	CGTGCGATCTCCAAGCACTC
	Reverse	CCAAGTTATCAGGGGTCCGA
B cell leukemia-2 (Bcl-2)	Forward	TTTGAGTTCGGTGGGGTCAT
	Reverse	GGCCGTACAGTTCCACGAAG
caspase-3	Forward	GAACAACGAAACCTCCGTGG
	Reverse	TCCCAGAGTCCATTGATTTGC
Interleukin-6 (IL-6)	Forward	AAGAAAACACCAGGGTCAGC
	Reverse	TGGTTTTTCTGCTGCAGGTTC
Tumor necrosis factor-α (TNF-α)	Forward	AGCCCACGTAGTAGCAAACC
	Reverse	TGAGTGAGGAGCACGTAGGA
Matrix metallopeptidase-13 (MMP-13)	Forward	TTTCTCGTTGCTGCCCATGA
	Reverse	GGGTGTTTAGGGTTGGGGTC
Manganese superoxide dismutase (MnSOD)	Forward	ACAAACCTGAGCCCTAACGG
	Reverse	AAGCGTGTTCCCACACATCA
Superoxide dismutase [Cu–Zn]-like (CuZnSOD)	Forward	GCCGCTGCGGAGTCAT
	Reverse	TCGGTCAGTCCTGTTATGCG
Autophagy related 5 (Atg5)	Forward	GACGACGACTGAACGACCTT
	Reverse	GAGCAATTGCGGAAGGACAC
Autophagy related 7 (Atg7)	Forward	TTCGACAAATGCACCGCTTG
	Reverse	ACTCAGACGGTCTCCTCGTC
Beclin-1	Forward	CCAGGTCAAACCAGGAGACC
	Reverse	CCCCGATCAGAGTGAAGCTG
Mechanistic target of rapamycin kinase (mTOR)	Forward	GGCAAGATGCTGGGGACC
	Reverse	CGCGTTGACTCTTCCTGACT
Alkaline phosphatase (ALP)	Forward	TCTCTGAGCCTCGTGAAACG
	Reverse	GCTCACCTCAGACACACCTC
Collagen type I (Col I)	Forward	CAGCGGCTCCCCATTTTCTA
	Reverse	ATCTCAGCTCGCATAGCACC
Osteocalcin (OCN)	Forward	AGAGTCTGGCAGAGGCTCA
	Reverse	CAGGGGATCCGGGTAAGGA
Osteopontin (OPN)	Forward	CACAGCGTGGAAACCCAAAG
	Reverse	TGGCCTCGCGCTTATATTGT
Peroxisome proliferator-activated receptor gamma (PPARγ)	Forward	TCACAAGAGGTGACCCAATG
	Reverse	CCATCCTTCACAAGCATGAA
Fatty acid binding protein 4 (FABP4)	Forward	TTCCTGTCGTCTGCGGTGATT
	Reverse	GATGCCTTTGTGGGAACCTGG
Collagen type II (Col II)	Forward	ACGCTCAAGTCGCTGAACAACC
	Reverse	ATCCAGTAGTCTCCGCTCTTCCAC
ACAN	Forward	CTGATCCACTGTCCAAGCACCATG
	Reverse	ATCCACGCCAGGCTCCACTC

#### Western blotting

Protein was extracted from cell samples using RIPA Lysis Buffer (Beyotime) containing phenylmethanesulfonyl fluoride (PMSF) (Beyotime, China). Equal protein quantities (60 µg) were denatured using SDS-PAGE loading buffer, separated by 6–15% polyacrylamide gel electrophoresis, and transferred to PVDF membranes (Millipore, Billerica, MA, USA). Prior to hybridization with antibodies, the blots were cut according to the weight of target proteins. Blots were probed at 4 °C overnight using primary antibodies against mTOR, p-mTOR, LC3, (1:1000, Cell Signaling Technology, Beverly, MA, USA), and GAPDH (1:10,000, Cell Signaling Technology). The blots were washed three times with PBST and then probed with an appropriate DyLight™ 800 4X PEG conjugated secondary antibody (1:10,000, Cell Signaling Technology) for 1 h. Protein bands were identified using an Odyssey Infrared Imaging System and protein levels were quantified relative to the GAPDH loading control using ImageJ software (NIH, Bethesda, Maryland, USA).

#### Statistical analysis

Data were presented as the mean ± SD and assessed using SPSS version 22.0. Parametric data were compared by one-way analysis of variance (ANOVA) with least significant difference (LSD) post-hoc tests, while non-parametric data were assessed by Mann–Whitney U tests. *p* values of < 0.05 were considered statistically significant.

## Results

### GABARAP increases BMSCs viability and proliferation by inhibiting intracellular ROS generation

**Figure 1:**
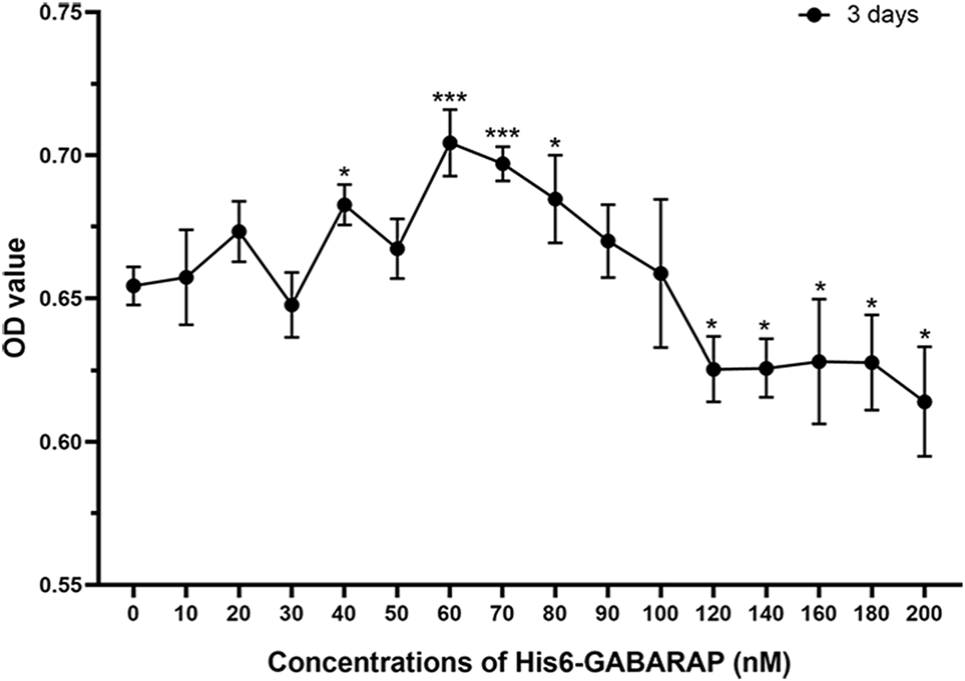
Edification the cytotoxicity of His6-GABARAP on BMSCs. MTT assay was applied after co-cultured with varying concentrations of His6-GABARAP for 3 days. Values are the means ± SD (*p < 0.05, ***p < 0.001 indicate the significant difference relative to the normal group, n = 3).

BMSCs were treated with varying concentrations of His6-GABARAP for 3 days, with no apparent toxicity observed for doses of 0–50 nM (Fig. 1). BMSCs exposed to 40, 60, 70, and 80 nM of His6-GABARAP displayed significantly higher proliferation, with the most dramatic increase seen for 60 nM; therefore, 30, 60, and 120 nM of His6-GABARAP were selected for the subsequent experiments.

**Figure 2:**
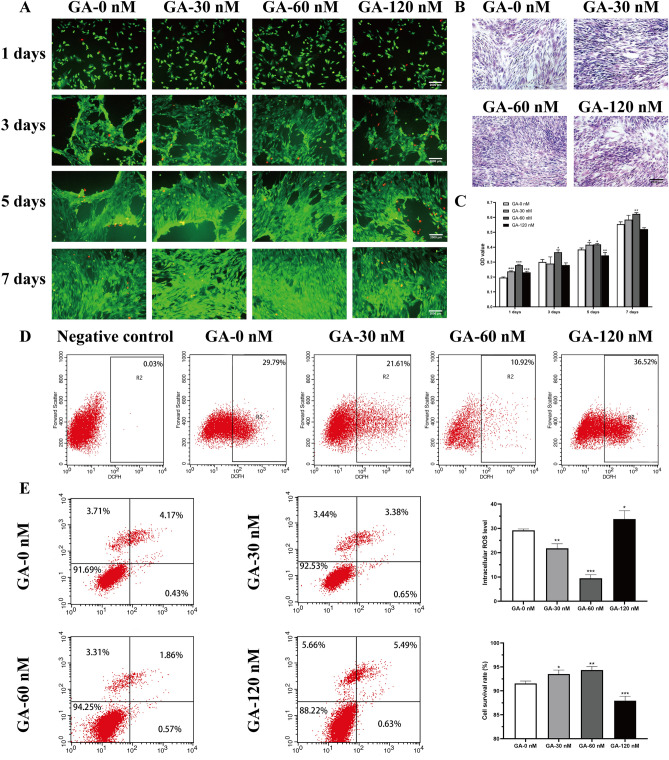
Promotion effects of His6-GABARAP on normal BMSCs. MTT assay (C) and FDA/PI staining (A) were applied to detect the cytotoxicity and viability of His6-GABARAP at days 1, 3, 5, and 7. Cells were treated with various concentrations of His6-GABARAP for 7 days. (B) H&E stained for cell morphology. (D) Flow cytometry for intracellular ROS. (E) Quantitative flow cytometry for viability. GA-0 nM (without His6-GABARAP); GA-30 nM (with 30 nM His6-GABARAP); GA-60 nM (with 60 nM His6-GABARAP); GA-120 nM (with 120 nM His6-GABARAP). Values are the means ± SD, n = 3. *p < 0.05, **p < 0.01, ***p < 0.001 relative to the normal group. Scale bar, 2000 µm.

First, we carried out HE staining to evaluate changes in cell morphology after His6-GABARAP treatment for 7 days. His6-GABARAP did not affect the typical fibroblastic cell morphology of cultured BMSCs but did increase the cell density compared to the normal group (Fig. 2B), as confirmed by MTT assays. As shown in Fig. 2C, His6-GABARAP concentrations of 30, 60, and 120 nM increased cell proliferation at days 1, with 60 nM of His6-GABARAP particularly increasing proliferation on days 3 (21.8 ± 5.2% higher than the control group), 5 (8.9 ± 1.4% higher than the control group), and 7 (12.4 ± 1.6% higher than the control group).

Next, we examined the effects of His6-GABARAP on BMSCs viability by Annexin V-FITC, PI, and FDA/PI staining. BMSCs treated with 30 and 60 nM of His6-GABARAP showed a significantly higher proportion of viable cells after 7 days of treatment (Fig. 2E), with the highest percentage of live cells observed in the 60 nM His6-GABARAP-treated group (94.4 ± 0.7%). Staining with FDA/PI dyes, which differentiate between dead and live cells, revealed that BMSCs exposed to 30 and 60 nM of His6-GABARAP showed significantly higher viability after 1, 3, 5, and 7 days of treatment (Fig. 2A), with the optimal effects observed at 60 nM. After 7 days, 30 and 60 nM of His6-GABARAP also decreased intracellular ROS levels to 21.8 ± 1.9 and 9.4 ± 1.5%, respectively (Fig. 2D). Taken together, these results demonstrate that GABARAP increases BMSCs viability by blocking intracellular ROS generation.

### GABARAP protects BMSCs against IL-1β-induced apoptosis, inflammation, and autophagy inhibition

**Figure 3:**
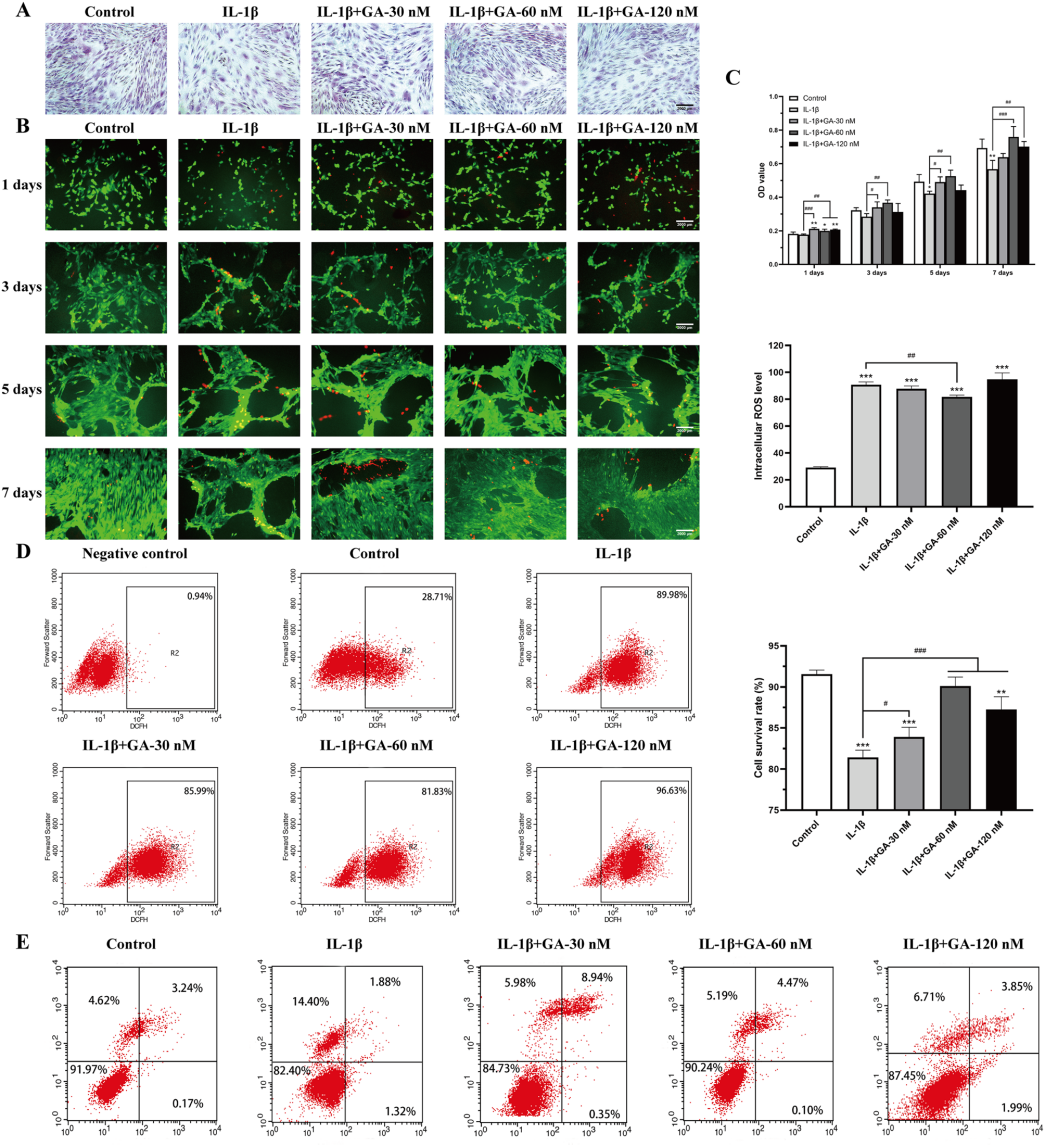
Protective effects of His6-GABARAP on IL-1β-induced BMSCs. H&E (**A**), FDA/PI staining (**B**), MTT assay (**C**), and Flow cytometry (**D**, **E**) were applied for cell morphology, viability, proliferation, and intracellular ROS. Control (without IL-1β); IL-1β (with 10 ng/mL IL-1β); IL-1β + GA-30 nM (with 10 ng/mL IL-1β and 30 nM His6-GABARAP); IL-1β + GA-60 nM (with 10 ng/mL IL-1β and 60 nM His6-GABARAP); IL-1β + GA-120 nM (with 10 ng/mL IL-1β and 120 nM His6-GABARAP). Values are presented as means ± SD, n = 3. *p < 0.05, **p < 0.01, ***p < 0.001 relative to the control group; #*p* < 0.05, ##*p* < 0.01, ###*p* < 0.001 relative to the IL-1β group. Scale bar, 2000 µm.

Next, we investigated the protective effects of His6-GABARAP in BMSCs treated with 10 ng/mL of IL-1β. When exposed to IL-1β alone, BMSCs displayed a flattened, rounded shape; however, the cells maintained a fibroblastic morphology when co-treated with His6-GABARAP (Fig. 3A). At a concentration of 60 nM, His6-GABARAP significantly increased the rate of cell proliferation to 12.6 ± 5.9, 29.5 ± 5.8, 24.7 ± 8.5, and 33.8 ± 10.9% higher than the IL-1β-treated group on days 1, 3, 5, and 7, respectively (Fig. 3C). Flow cytometry (Fig. 3E) and FDA/PI (Fig. 3B) staining also showed that His6-GABARAP treatment could increase the percentage of live cells and decrease the level of apoptosis induced by IL-1β. Similarly, His6-GABARAP was able to reverse the up-regulation of the pro-apoptotic gene caspase-3 in IL-1β-treated cells (Fig. 5A) and increased the gene expression ratio of Bcl-2/BAX (Fig. 5B-D). Moreover, His6-GABARAP-treated cells displayed dramatically lower ROS levels than the IL-1β-treated group (Fig. 3D).

**Figure 4:**
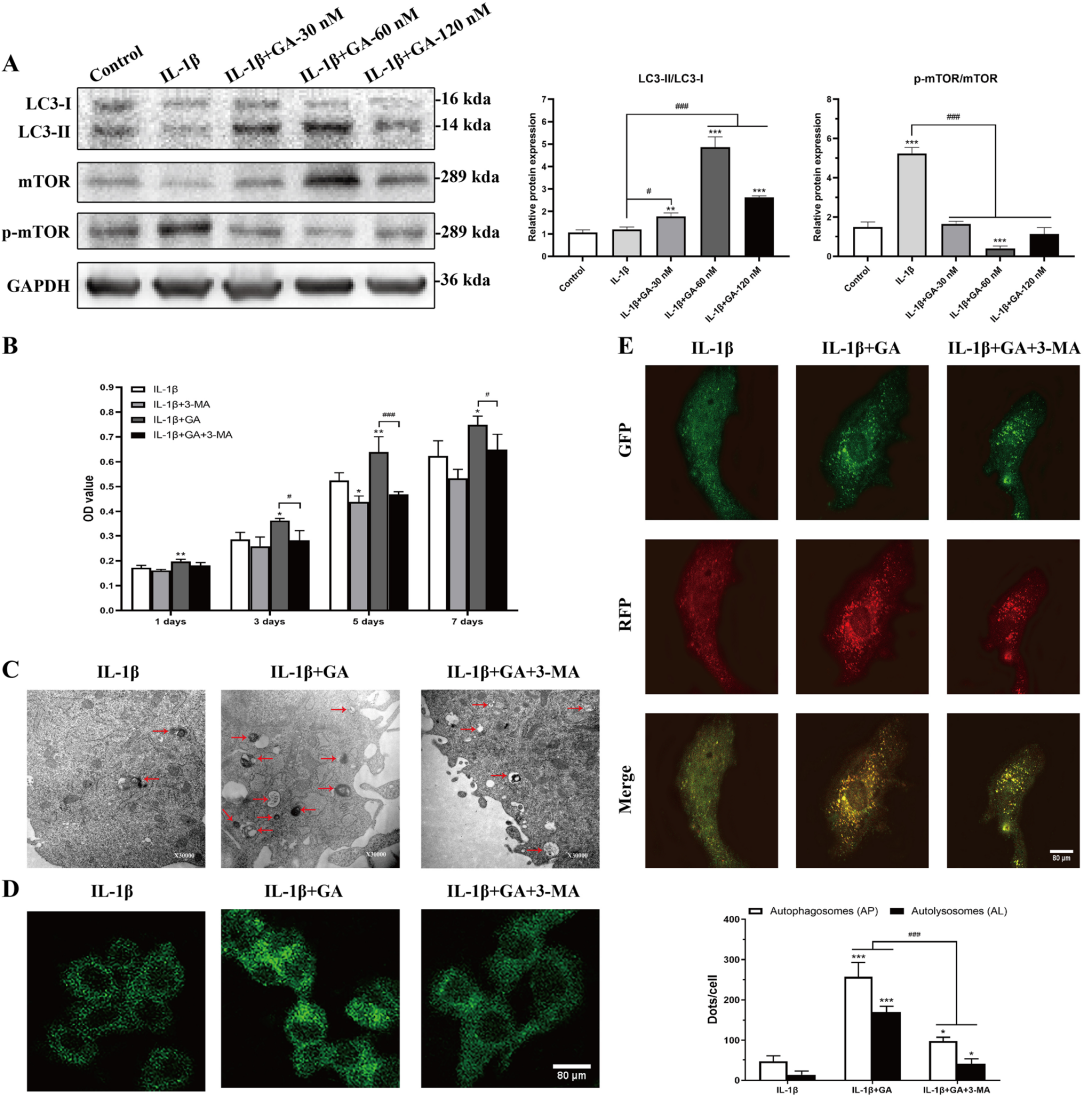
Effects of His6-GABARAP on autophagic flux in BMSCs in vitro. (**A**) Western blot of LC3, mTOR, and p-mTOR in different groups were detected respectively and the semi-quantitative analysis of LC3 II/I ratio and mTOR/p-mTOR are shown. (**B**) MTT assay were applied to detect the cell proliferation at days 1, 3, 5, and 7. (**C**) TEM images of the autophaghic change in BMSCs. Single arrow: autophagolysosome and autophagosome with double membrane structure. Original magnification × 30,000. (**D**) Representative pictures of MDC staining. Scale bar, 80 μm. (**E**) Representative pictures of immunofluorescent BMSCs expressing mRFP-GFP-LC3. GFP dots are green, and mRFP dots are red. Scale bar, 80 μm. And Semi-quantitative analysis of autophagosomes (AP; yellow dots in merged images) and autolysosomes (AL; red only dots in merged images). Control (without IL-1β); IL-1β (with 10 ng/mL IL-1β); IL-1β + 3-MA (with 10 ng/mL IL-1β and 3-MA); IL-1β + GA (with 10 ng/mL IL-1β and 60 nM His6-GABARAP); IL-1β + GA + 3-MA (with 10 ng/mL IL-1β, 60 nM His6-GABARAP, and 3-MA). Values are presented as means ± SD, n = 3. *p < 0.05, **p < 0.01, ***p < 0.001 relative to the control group; #*p* < 0.05, ##*p* < 0.01, ###*p* < 0.001 relative to the IL-1β group.

**Figure 5:**
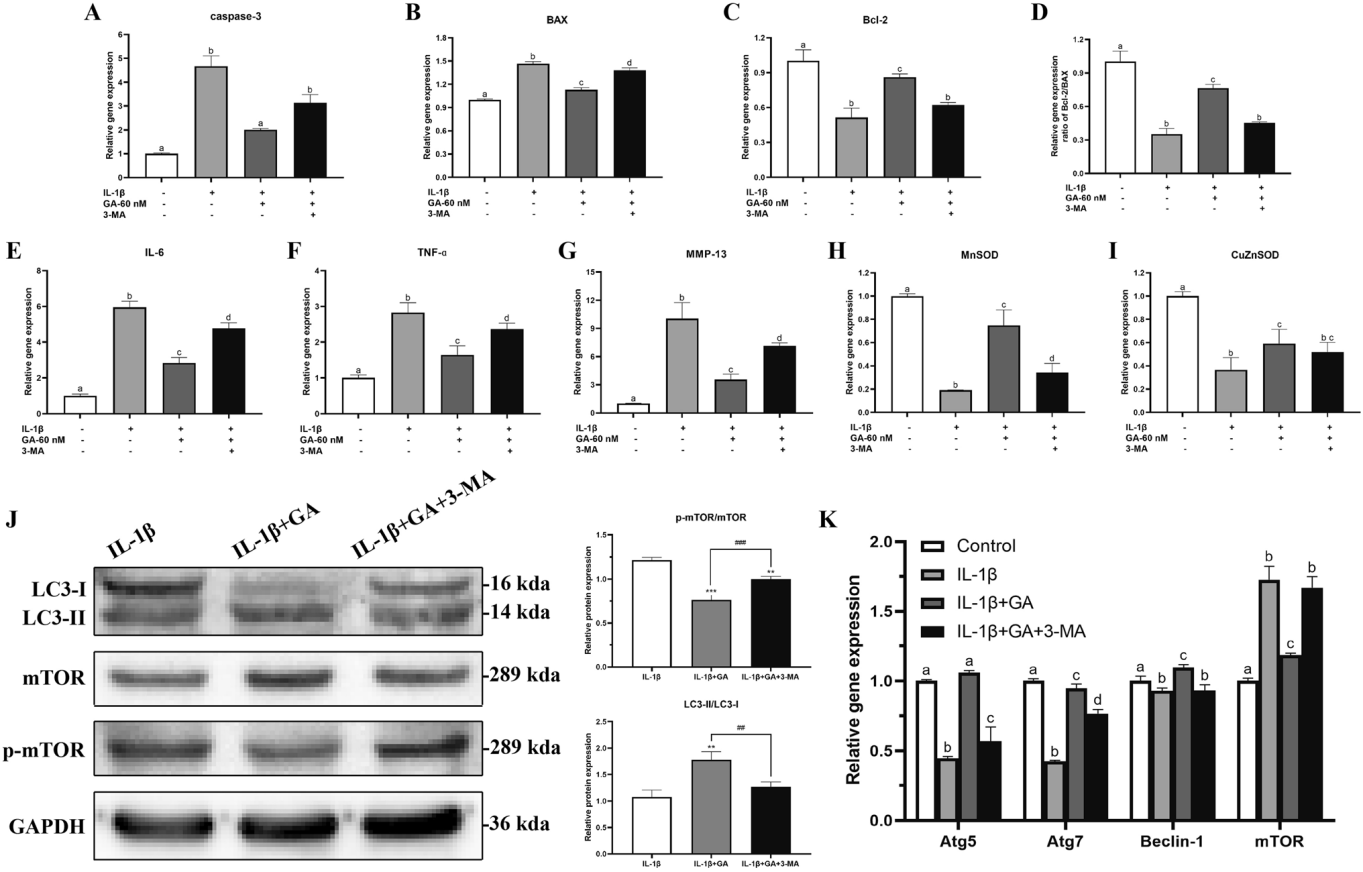
Effect of His6-GABARAP on the treatment of IL-1β-induced BMSCs. (A-I, and K) qRT-PCR was used to analyze the gene expression levels of caspase-3, BAX, Bcl-2, Bcl-2/BAX ratio, TNF-α, IL-6, MMP-13, MnSOD, CuZnSOD, Atg5, Atg7, Beclin-1, and mTOR in vitro. (J) Western blot was used to analyze the protein expression of LC3, mTOR, and p-mTOR. Values are presented as means ± SD, n = 3. Bars with different letters are significantly different from each other at *p* < 0.05 and those with the same letter exhibit no significant difference. **p < 0.01, ***p < 0.001 relative to the IL-1β group; ##*p* < 0.01, ###*p* < 0.001 relative to the IL-1β + GA group.

To determine whether His6-GABARAP functioned by inhibiting inflammation, we performed qRT-PCR to detect the expression levels of inflammatory factors such as IL-6 (Fig. 5E), TNF-α (Fig. 5F), and MMP-13 (Fig. 5G). Compared to the vehicle-treated cells, His6-GABARAP treatment effectively suppressed the IL-1β-induced increase in inflammatory cytokine levels and restored the expression of antioxidant-specific markers including MnSOD (Fig. 5H) and CuZnSOD (Fig. 5I) in BMSCs. To investigate whether His6-GABARAP triggered autophagy in BMSCs under inflammatory conditions, we measured Atg5, Atg7, LC3, Beclin-1, and mTOR expression levels by qRT-PCR and western blotting, finding that LC3-II/LC3-I, Atg5, Atg7, and Beclin-1 expression levels were dramatically lowered by IL-1β treatment and reversed when co-cultured with His6-GABARAP (Figs. 4A and 5K). Meanwhile, the expression of mTOR was suppressed by His6-GABARAP as compared with IL-1β-treated group (Figs. 4A and 5K). Taken together, these results demonstrate that GABARAP alleviates pathological changes in BMSCs by blocking intracellular ROS generation, inflammation, and apoptosis, and that these effects are likely mediated via autophagy activation.

### GABARAP up-regulates cell survival by targeting autophagy mechanisms

To clarify the role of autophagy in the protective effects of His6-GABARAP in BMSCs viability and inflammation, we treated IL-1β-induced BMSCs with His6-GABARAP with or without 3-MA. MTT assays confirmed that 3-MA negatively affected the proliferation of IL-1β-treated BMSCs and effectively blocked the promotion of cell proliferation by His6-GABARAP (Fig. 4B). By detecting the expression of apoptosis-, antioxidant-, and inflammation-related cytokines in BMSCs, we found that 3-MA prevented the His6-GABARAP-mediated inhibition of cell apoptosis, oxidative stress, and inflammation (Fig. 5A–I), indicating that 3-MA treatment has an inhibitory effect on BMSCs viability that is promoted by His6-GABARAP in an inflammatory microenvironment.

To explore the role of autophagy in the interaction between 3-MA and His6-GABARAP, we detected the autophagy-related cytokines Atg5, Atg7, LC3, Beclin-1, and mTOR. As shown in Fig. 5J and 5K, 3-MA decreased Atg5, Atg7, and Beclin-1 expression levels and the LC3-II/LC3-I ratio which were increased by His6-GABARAP, along with the number of MDC-labeled autophagic vacuoles in IL-1β-stimulated BMSCs (Fig. 4D). mTOR expression levels were again elevated by 3-MA as compared with His6-GABARAP-treated group (Fig. 5J and 5K). However, the downregulation of autophagy markers and the presence of numerous autophagic vacuoles are static inhibitors of autophagy. Thus, to detect autophagic flux we transduced BMSCs with RFP-GFP–LC3 to simultaneously track autophagosomes (yellow punctae that are a combination of red and green fluorescence) and autophagolysosomes (red punctae). His6-GABARAP treatment significantly increased the proportion of autophagolysosomes in the cytoplasm of IL-1β-treated BMSCs (Fig. 4E), indicating that autophagy was induced. In addition, TEM confirmed the increased number of autophagic vesicles and autolysosomes in the His6-GABARAP-treated BMSCs (Fig. 4C). As expected, 3-MA partially inhibited the autophagic flux and autophagolysosome-inducing effects of His6-GABARAP (Fig. 4C and E). Taken together, these findings suggest that His6-GABARAP activates autophagic flux in IL-1β-treated BMSCs as the underlying basis of its regenerative effects.

### GABARAP activation modulates chondrogenic differentiation and adipogenesis in vitro

**Figure 6:**
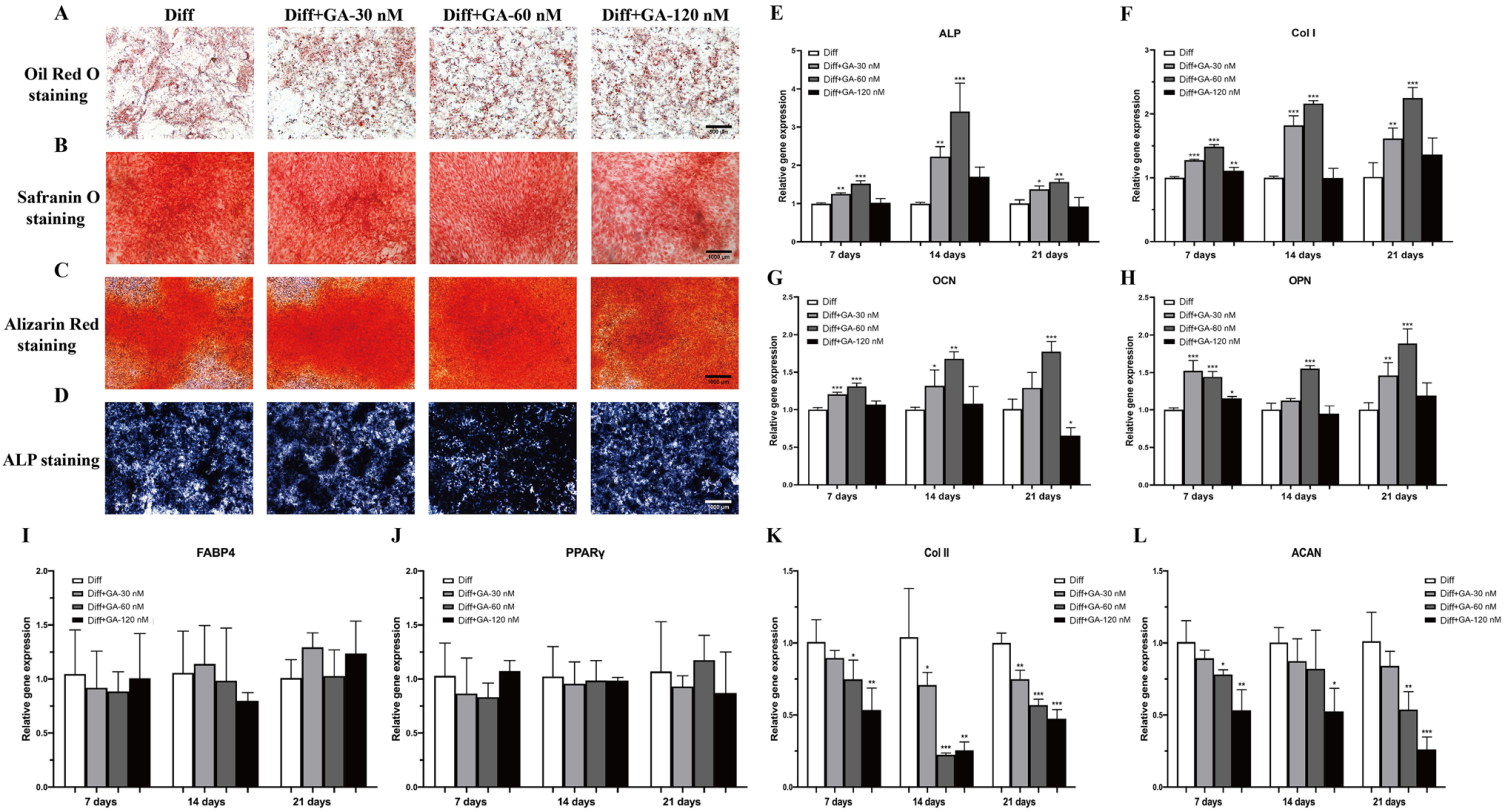
The effect of His6-GABARAP on BMSCs differentiation. Adipogenesis and chondrogenesis differentiation of BMSCs were verified by Oil Red O (**A**) and safranin O (**B**) staining, respectively, in 21 days. Qualitative assessment of calcium deposition in BMSCs was stained by Alizarin Red (**C**) and ALP (**D**) staining in 21 days. qRT-PCR was used to analyze the gene expression levels of ALP (**E**), Col I (**F**), OCN (**G**), OPN (**H**), FABP4 (**I**), PPARγ (**J**), Col II (**K**), and ACAN (**L**) during differentiation. Diff (with corresponding differentiation); Diff + GA-30 nM (with corresponding differentiation and 30 nM His6-GABARAP); Diff + GA-60 nM (with corresponding differentiation and 60 nM His6-GABARAP); Diff + GA-120 nM (with corresponding differentiation and 120 nM His6-GABARAP). Values are the means ± SD, n = 3. *p < 0.05, **p < 0.01, ***p < 0.001 relative to the Diff group. Scale bar, 500&1000 µm.

BMSCs can differentiate into multiple cell types, including chondrocytes and adipocytes; therefore, to determine the effect of His6-GABARAP on BMSCs differentiation, we cultured MSCs in chondrogenesis or adipogenesis medium. As verified by very low levels of regulation in the Oil Red O stain, His6-GABARAP did not affect BMSCs adipogenesis (Fig. 6A). Meanwhile, the expression level of adipogenic differentiation-connected genes, including FABP4 and PPARγ, also showed that His6-GABARAP had little effect on BMSCs adipogenesis (Fig. 6I and J). However, the results of Safranin O staining demonstrated that His6-GABARAP inhibited chondrocyte formation (Fig. 6B). At the genetic level, genes expression level of chondrogenesis markers Col II and ACAN were significantly decreased by His6-GABARAP (Fig. 6K and L), which demonstrated that chondrogenesis differentiation was markedly reduced in His6-GABARAP-treated BMSCs compared to WT differentiated BMSCs. These results may provide evidence for the additional role of His6-GABARAP in BMSCs chondrogenesis and adipogenesis.

### GABARAP maintains BMSC osteogenic differentiation in normal and inflammatory microenvironments

Osteogenic differentiation was induced by culturing BMSCs in osteogenic media with or without 30, 60, and 120 nM of His6-GABARAP. As shown in Fig. 6C and D, ALP and Alizarin Red staining after 21 days indicated that His6-GABARAP stimulated BMSCs osteogenesis, with 60 nM of His6-GABARAP causing the most significant change in calcium deposition. We then quantified the mRNA levels of the osteoblast marker genes Col I, ALP, OCN, and OPN after 7, 14, and 21 days (Fig. 6E–H). His6-GABARAP increased Col I, OPN, ALP, and OCN transcription, particularly at a treatment concentration of 60 nM His6-GABARAP. Therefore, we used 60 nM of His6-GABARAP for subsequent experiments.

**Figure 7:**
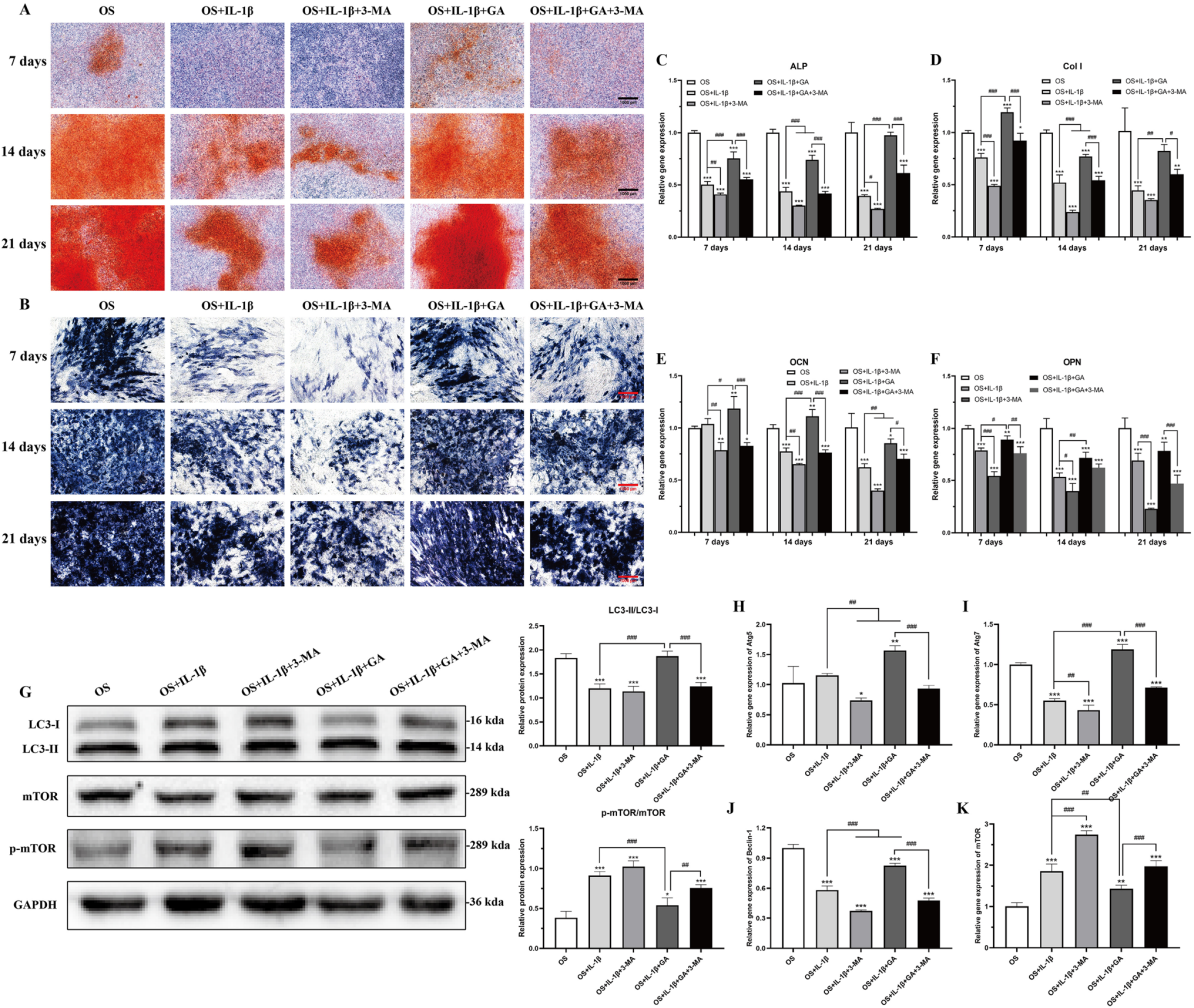
The effect of His6-GABARAP on IL-1β-induced BMSCs osteogenic differentiation. Qualitative assessment of calcium deposition in BMSCs was stained by Alizarin Red (**A**) and ALP (**B**) staining after 7, 14, and 21 days of osteogenic differentiation culture. (**C**–**F**, and **H**–**K**) qRT-PCR was used to analyze the gene expression levels of ALP, Col I, OCN, OPN, Atg5, Atg7, Beclin-1, and mTOR during osteogenic differentiation. (**G**) Western blot was used to analyze the protein expression of LC3, mTOR, and p-mTOR. OS (with osteogenic differentiation); OS + IL-1β (with osteogenic differentiation and 10 ng/mL IL-1β); OS + IL-1β + 3-MA (with osteogenic differentiation, 10 ng/mL IL-1β, and 3-MA); OS + IL-1β + GA (with osteogenic differentiation, 10 ng/mL IL-1β, and 60 nM His6-GABARAP); OS + IL-1β + GA + 3-MA (with osteogenic differentiation, 10 ng/mL IL-1β, 60 nM His6-GABARAP, and 3-MA). Values are presented as means ± SD, n = 3. *p < 0.05, **p < 0.01, ***p < 0.001 relative to the OS group; #*p* < 0.05, ##*p* < 0.01, ###*p* < 0.001 relative to the OS + IL-1β group. Scale bar, 1000 µm.

In the presence of IL-1β, 60 nM of His6-GABARAP stimulated denser deposition of Alizarin Red and ALP staining after 7, 14, and 21 days of osteogenic differentiation culture (Fig. 7A and B). Moreover, IL-1β-treated BMSCs had dramatically lower mRNA expression of the osteoblastic differentiation factors Col I, ALP, OCN, and OPN (Fig. 7C–F); however, treatment with 60 nM of His6-GABARAP yielded higher COL I, ALP, OCN, and OPN mRNA expression levels than in IL-1β treated BMSCs. Taken together, these results suggest that His6-GABARAP can maintain their BMSCs osteogenic differentiation under inflammatory conditions.

### Autophagy plays a role in GABARAP-promoted BMSCs osteogenic differentiation

To determine the mechanisms responsible for the increased osteoblastogenesis observed in His6-GABARAP-treated BMSCs under inflammatory conditions, we used 3-MA to suppress autophagy. Interestingly, inhibiting autophagy with 3-MA enhanced the inhibitory effects of IL-1β in BMSCs osteogenic differentiation and abrogated the restorative effects of His6-GABARAP in vitro (Fig. 7A–F). Similarly, culturing BMSCs with His6-GABARAP in presence of IL-1β under osteogenic differentiation significantly increased the gene and protein expression of Atg5, Atg7, Beclin-1, and the ratio of LC3-II/LC3-I (Fig. 7G–J). Moreover, BMSCs co-cultured with 3-MA and His6-GABARAP displayed significantly lower levels of autophagy activation, as evidenced by downregulated Atg5, Atg7, Beclin-1, and LC3-II/LC3-I expression levels and upregulated expression level of mTOR (Fig. 7G–K). Thus, these results suggest that autophagy activation plays a critical role in His6-GABARAP promoting BMSCs osteogenic differentiation.

## Discussion

Previously, study reported that weekly intra-articular His6-GABARAP administration effectively promotes the therapeutic effect of BMSC-derived chondrocytes in OA lesions^38^; however, the initiation and progression of OA involves a variety of pathological mechanisms, including the loss of bone mass in subchondral bone^39,40^ and synovium lesions^41^. Meanwhile, its specific therapeutic mechanisms, particularly the associations between GABARAP and BMSCs or chondrocytes under inflammatory conditions, remain unknown. In present study, it had been demonstrated that autophagy plays an important role in promoting BMSCs osteogenic differentiation and restricts intracellular ROS generation^27^. Furthermore, it was also revealed that autophagy activation could enhance BMSCs proliferation^42^. Consistently, our pilot study also found that GABARAP could mediate BMSCs cell viability and osteogenic differentiation, which are inhibited by the pro-inflammatory factor IL-1β; thus, it may provide a potential application of GABARAP in OA repairment by promoting subchondral bone remodeling.

The induction of cellular ROS generation and activation of apoptosis signaling by IL-1β has been increasingly recognized to play a vital role in cell stress^43,44^, and followed to induce senescence and apoptosis in various cell types, including BMSCs^6^, chondrocytes^45^, and osteocytes^46^, thus, IL-1β with significant pathological implications in the progression of many inflammatory diseases. Consistently, we found that IL-1β in the microenvironment may serve as an important modulator of BMSCs viability and proliferation and increase the expression of the inflammatory markers IL-6, TNF-ɑ, and MMP-13. However, His6-GABARAP treatment effectively maintained cell survival, restored the fibroblastic-like morphology of BMSCs, and downregulated pro-inflammatory cytokines and apoptosis-related markers. Furthermore, GABARAP not only suppressed inflammatory damage in BMSCs, but also enhanced BMSC proliferation and viability without IL-1β.

Intracellular ROS generation is a major pathological driver of apoptosis in BMSCs^47^, which in turn plays a vital role in triggering inflammation^48,49^. Studies have demonstrated that autophagy plays a key role in suppressing ROS formation^50^; therefore, we examined the effect of GABARAP on ROS generation in BMSCs. GABARAP stimulation significantly decreased ROS levels in BMSCs and the effect was maintained when co-cultured with IL-1β. In addition, His6-GABARAP treatment increased the expression of MnSOD and CuZnSOD, which are antioxidant factors in the superoxide dismutase pathways^51^.

Autophagy is a catabolic process wherein damaged proteins and organelles are phagocytosed and degraded to maintain energy levels and support organelle renewal^52^. Under stress conditions such as hypoxia and starvation, activation of autophagy can prevent apoptosis and maintain cellular homeostasis^53–55^. Indeed, we found that autophagic flux was significantly suppressed in IL-1β-induced BMSCs and restored by His6-GABARAP treatment. The phosphoinositide 3-kinase (PI3K) blocker 3-MA, which is routinely used to inhibit autophagy^56^, markedly abrogated the effects of GABARAP on apoptosis, the inflammatory response, and ROS generation. Therefore, our findings suggest that GABARAP promotes BMSCs proliferation and reduces inflammation-induced apoptosis by activating autophagy; however, more studies are required to clarify the mechanism further.

The capacity to differentiate into several cell lines is a crucial characteristic of BMSCs, and it has been reported that the stimulation of intracellular ROS degradation by autophagy activation plays a vital role in regulating BMSCs osteogenic differentiation^27^. In this study, we observed that the pharmacological induction of GABARAP was closely associated with BMSCs osteogenesis and calcium deposition. Due to the importance of IL-1β in MSCs differentiation, multiple studies have demonstrated that IL-1β plays a crucial role in suppressing osteogenesis^57^ and upregulating metalloproteinase expression to degrade the extracellular matrix of bone tissue^18^. Conversely, studies have also suggested that IL-1β stimulation promotes calcium deposition and endochondral ossification^58,59^; however, recent studies have shown that the effects of IL-1β on stem cells is dependent upon the target cell line and the IL-1β concentration^36^. Notably, in our study BMSCs displayed a lower osteoblast capacity when incubated with 10 ng/mL of IL-1β, with His6-GABARAP treatment improving osteogenic differentiation in the presence of IL-1β via a mechanism closely related to the up-regulation of autophagy markers. As hypothesized, 3-MA stimulation effectively inhibited the effect of GABARAP, suggesting that GABARAP stimulates the osteogenic differentiation of IL-1β-induced BMSCs by activating autophagy. This stimulatory effect of GABARAP may be due to the role of autophagy maintenance in ROS generation, since autophagy has been shown to be a good candidate for maintaining the redox homeostasis of BMSCs^27^. Moreover, elevated ROS levels in BMSCs have been reported to modulate osteogenesis, with ROS scavenging able to effectively restore their osteogenic capacity^60^. However, the specific mechanisms during osteogenic differentiation, particularly in the presence of GABARAP, require further exploration. Taken together, we showed that GABARAP promotes osteogenic differentiation in IL-1β-induced BMSCs, at least partly by activating the autophagy pathway.

In conclusion, GABARAP improves BMSCs proliferation and partially protects cell against IL-1β-induced inflammation and intracellular ROS generation. In addition, our findings suggest that GABARAP promotes the osteogenic differentiation of BMSCs exposed to IL-1β and that autophagy activation is involved in its effect on BMSCs viability and osteogenic differentiation. Further studies will be conducted focusing on the mechanisms underlying the effects of GABARAP under more complicated inflammatory conditions. However, GABARAP may represent a novel therapeutic option for treating inflammation in OA and an effective method for promoting bone tissue regeneration.

## Supplementary Information

Supplementary Information.

## Data Availability

The data used to support the findings of this study are available from the corresponding author upon request.
