# Supplementary material for: GABARAP ameliorates IL-1β-induced inflammatory responses and osteogenic differentiation in bone marrow-derived stromal cells by activating autophagy
Source: Sci Rep. 2021 Jun 2;11:11561. doi: 10.1038/s41598-021-90586-9 (PMC8172545; doi:10.1038/s41598-021-90586-9)
Supplement: Supplementary file 1 — Supplementary Information. [file 41598_2021_90586_MOESM1_ESM.pdf]

Supplementary information

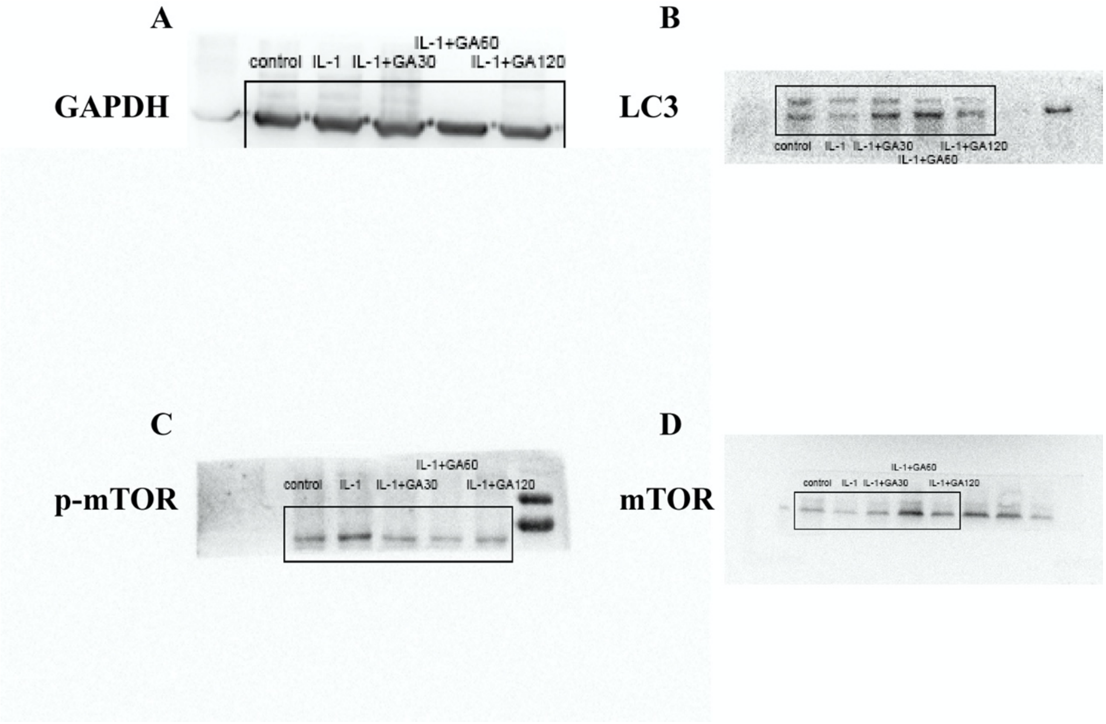

Supplementary Figure 1: Original western blot membranes for membrane strips presented in Figure 4 of the manuscript.

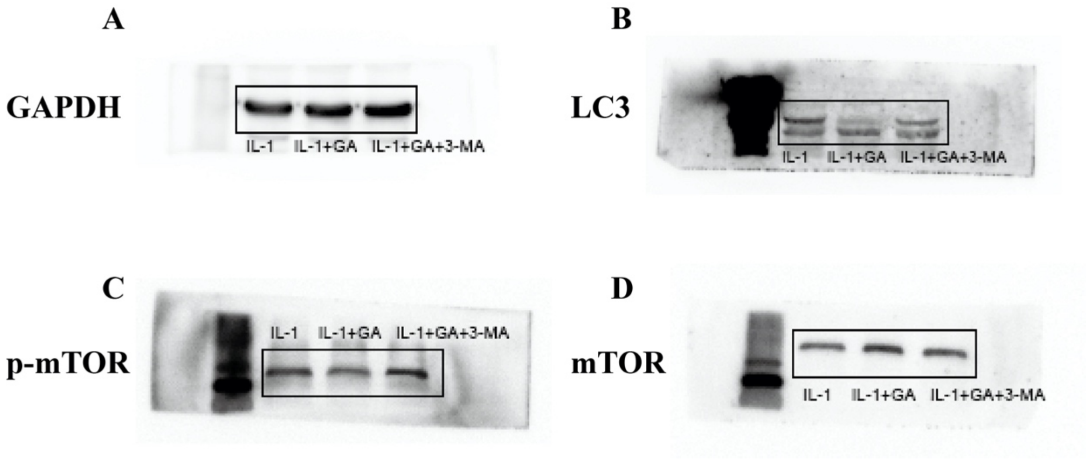

Supplementary Figure 2: Original western blot membranes for membrane strips presented in Figure 5 of the manuscript

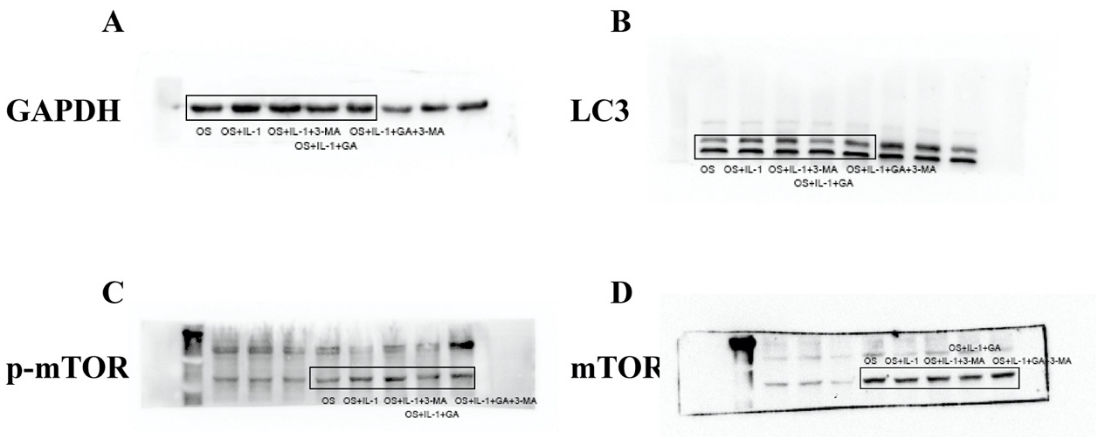

Supplementary Figure 3: Original western blot membranes for membrane strips presented in Figure 7 of the manuscript
